# Patellar dislocation due to iatrogenic quadriceps fibrosis: results of operative treatment in 54 cases

**DOI:** 10.1007/s11832-014-0564-5

**Published:** 2014-02-08

**Authors:** Nguyen Ngoc Hung, Do Tan, Nguyen Do Ngoc Hien

**Affiliations:** 1 Vietnam National Hospital of Pediatrics, 18/879 La Thanh Road, Dong Da District, Hanoi, Vietnam; 2 Hanoi Medical University, Hanoi, Vietnam

**Keywords:** Patellar instability, Patellar dislocation, Iatrogenic quadriceps fibrosis, Surgical treatment, Quadricepsplasty, Capsulorrhaphy, Subluxation of the patella, Developmental dysplasia of the patella (DDP), Malformative dislocation

## Abstract

**Objective:**

To evaluate the clinical and functional results of a surgical treatment of patellar dislocation whose etiology was iatrogenic quadriceps fibrosis in children.

**Materials and methods:**

A prospective study was undertaken from February 2004 to December 2009. The study included 54 pediatric patients (56 knees) that had developed dislocation of the patella after repeated intramuscular injections of antibiotic(s) into the quadriceps muscle. There were 11 males (20.4 %) and 43 females (79.6 %). The patients’ mean age at surgery was 7 years, 9 months (range 6 years, 4 months to 12 years, 6 months). A complete history of each patient was recorded. The affected knees were evaluated preoperatively and postoperatively on the basis of the symptoms, signs, and roentgenographic findings. Patellar dislocation was classified according Bensahel’s criteria. All patients had a three-part surgical procedure that combined capsulorrhaphy, quadricepsplasty, and transfer of the vastus medialis oblique to the superior border of the patella.

**Results:**

There has been no poor postsurgical result or recurrence so far; we have noted an ugly scar in nine knees (16.1 %), limitation of the knee flexion in five knees (8.9 %), and loss of extension of 5 °–20 ° in four knees (7.1 %). Overall, we attained excellent results in 39 knees (69.7 %), good results in 13 knees (23.2 %), and fair results in four knees (7.1 %).

**Conclusion:**

In our cases of pediatric dislocation of the patella caused by iatrogenic quadriceps fibrosis, the introduced three-part surgical procedure has shown great success in restoring the realignment mechanism of the patella. The technique is simple, safe, and effective in skeletally immature children.

## Introduction

Patellar dislocation is common in children, and many operations have been described for its treatment [1–3]. Surgical treatments include both proximal and distal realignment of the extensor mechanism, and most involve considerable surgical trauma, a large scar, and prolonged rehabilitation. Many factors contribute to patellar dislocation, including (1) congenital mechanisms (i.e., generalized ligamentous laxity, dysplasia of the patella or the femoral condyles, genu valgum, tight lateral bands, and patella alta [1–3]) and (2) a secondary mechanism, due to iatrogenic quadriceps fibrosis after intramuscular antibiotic(s) injections changed vector pull quadriceps, and fibrous vastus lateralis, vastus intermedius, and lateral retinacular patella; degeneration and contracture of the rectus femoris and vastus medialis.

This second etiology, patellar dislocation secondary to intramuscular injections, was the subject of our study. In 1961, Hnĕvkovský [4] first stimulated an interest in this etiology by his report of progressive fibrosis of the vastus intermedius in young children who had received intramuscular antibiotic injections. Gunn [5] demonstrated a causal relationship between intramuscular injections and quadriceps contracture, which, in turn, led to the habitual dislocation of the patella. Although all the conditions we now associate with this contracture are well known, the importance of the underlying muscle condition in each case has not been stressed.

Since 2004, our surgical team has treated dislocation of the patella in children using a three-part surgical procedure that combines capsulorrhaphy and quadricepsplasty with transferring the vastus medialis oblique (VMO) to the superior border of the patella. This paper’s objective was to evaluate the clinical and functional results of a surgical treatment of patellar dislocation whose etiology was iatrogenic quadriceps fibrosis in children. This technique has the benefits of simplicity and effectiveness in restoring the realignment mechanism of the patella.

## Materials and methods

### Patient selection criteria and information

A prospective study was conducted from February 2004 to December 2009. Fifty-four pediatric patients with 56 lateral dislocations of the patella (two bilateral cases, for a total of 56 knees) with a documented past history of multiple intramuscular injections during the neonatal period and patellar dislocation due to fibrous quadriceps were recruited. There were 11 males (20.4 %) and 43 females (79.6 %) in the study. The mean age of the patients at surgery was 7 years, 9 months. Most of the patients’ injections had been done during the first 24 months of life. The medical agents that had been injected were all antibiotics: penicillin, gentamicin, lincomycin, streptomycin, cloxacillin, etc. In these patients, there was no history of trauma or family history of patellar dislocation, genu valgum, or other congenital diseases. All patients had had at least three complete lateral dislocations of the patella; the patella had been seen or felt to lie on the lateral aspect of the femur.

Twenty-four right knees (42.9 %) and 32 left knees (57.1 %) were affected; two patients (2.7 %) had bilateral problems. Four knees (7.1 %) had persistent patellar dislocation, in which the patella rested laterally at all times, and the remaining 52 knees (92.9 %) had recurrent patellar dislocation. The cardinal physical sign was the lateral dislocation of the patella each time the knee flexed. With the patella held firmly in the femoral groove, we saw a limitation of knee flexion of between 10° and 20° in 34 knees, and of 30° in 22 knees. When the patella had dislocated, full flexion was possible. Palpable bands attaching to the lateral border of the patella were noted in all knees. There were three patients with opposite knee stiffness and four patients with other joint stiffness caused by the post-injection of antibiotic(s) into fibrous muscles (elbow in one patient; abduction of the scapula in three other patients).

#### Exclusions

We excluded patients from the study who had undergone a previous operation for patellar instability or an internal derangement; had suffered from major trauma to the knee; or had neuromuscular disease, Down syndrome, or other congenital conditions, such as arthrogryposis multiplex congenita.

### Clinical and imaging examinations

Patellar dislocation was determined based on the clinical examination results and imaging findings as described below.

#### Clinical examination

For a dislocation, we would expect to find the following clinical signs: (a) knee deformation; (b) atrophy of the thigh compared to the opposite healthy thigh; (c) on knee flexion, a prominent lateral knee; patellar medial glide test <5 mm at 30° of knee flexion [6]; and (d) the patient being unable to run.

We also tested the following factors: Q-angle [7], genu valgum, and the ligament laxity.

### Imaging findings

Each patient had radiography according to Hughston [38], magnetic resonance imaging (MRI), computed tomography (CT) scan, and ultrasound for the knee and thigh.

#### Roentgenogram (X-ray)

Patella alta: was diagnosed in the lateral view using the method of Insall and Salvati [8].

Lateral patellar tilt: in the axial view, we measured the lateral patellofemoral angle according to Laurin et al. [9].

Abnormal tilt: the abnormal tilt was also calculated in the axial view according to Grelsamer et al. [10].

#### MRI

Shallow patellar groove: the trochlear depth was assessed according to Seil et al. [11].

#### CT scan

Flattening of the distal lateral femoral condyles according to Dejour [12]: performing a CT scan of the distal femur sulcus lines were displayed in both the horizontal and sagittal planes.

- Normal trochlea: tip of lateral facet > sulcus line < tip of medial facet.
- Flat trochlea (crossing sign): tip of lateral facet = sulcus line = tip of medial facet.
- Lateral convexity (double contour): tip of lateral facet = sulcus line > tip of medial facet [12].

#### Ultrasound

We performed ultrasound of the muscles at the distal thigh to evaluate the presence of fibrous muscles, which demonstrated fibrous muscles in the distal third of the thigh in all patients.

### Classification of patellar dislocation

Our patients were classified according to Bensahel et al.’s criteria [13]:

- Type 1: Dislocation of the patella without major radiographic abnormality.
- Type 2: Dislocation of the patella with major patellofemoral dysplasia, namely a patella alta and a flat or convex trochlea.

### Conservative treatment

Conservative treatment was tried on patients. (In the first 6 weeks, the conservative treatment was performed with physiotherapy daily for 1 h. The physiotherapist undertook a passive movement with flexion and extension by holding the patella in the intercondylar groove and, furthermore, mobilized the knee in a superior–inferior and medial–lateral way.) After physical therapy, the knee was placed in a plastic splint with it flexed a maximum amount but without patellar dislocation. The knee flexion was gradually increased.

Conservative treatment was considered to have failed if the patient had recurrent patellar dislocation when the knee was flexed; surgery was then indicated for these children. Any patients who had experienced recurrent patellar dislocation were indicated immediately for surgery without conservative treatment.

### Surgical technique and procedure

A single surgeon (NNH) performed all of the patient operations. All pertinent clinical and operative records were prospectively reviewed.

#### Patient positioning

**Fig. 1:**
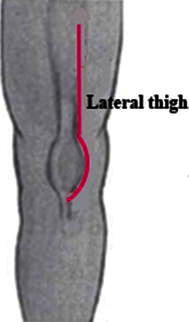
Skin incision

The patient was positioned supine on a standard operating table, placed under general anesthesia, with a sandbag behind the knee to keep it in between 5° and 10° of flexion; the anterolateral part of the thigh was exposed. We used the anterolateral approach that extended from the mid-third of the thigh to the tibial tuberosity, and a slightly curved lateral border of the patella (Fig. 1). The subcutaneous tissues were undermined sufficiently to create a skin flap that would allow us to expose the quadriceps muscle, the medial and lateral retinaculum of the patella, the iliotibial tract, the patella, and the patellar tendon. This incision avoided penetration into the capsule and the infrapatellar nerve.

The operation was performed in three procedural stages.

##### Procedure stage 1: releasing the lateral retinaculum and restoring the medial retinaculum

**Fig. 2:**
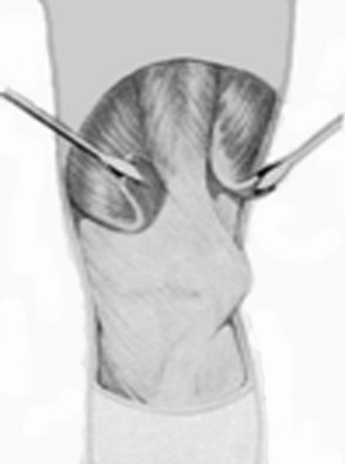
Detaching the insertion of the vastus lateralis and vastus medialis from the patella

The tight lateral bands were released from the patella and the incision continued proximally, lateral to the rectus femoris tendon, thus fully releasing the vastus lateralis and vastus medialis. A slightly curved longitudinal incision was made from the superolateral aspect of the patella proximally for 8 cm or more, as necessary. Any abnormal attachments of the iliotibial tract to the patella and to the lateral capsule were incised longitudinally. However, this procedure alone could not allow the stable reduction of the patella in the fully flexed knee. It was always necessary to detach the insertion of the vastus lateralis from the patella, separating it from the rectus femoris medially and the iliotibial tract laterally, then mobilizing it proximally (Fig. 2).

For dividing the tendon of the VMO, a well-defined margin of tendon must be retained on the muscle to permit its subsequent transfer to the superior border of the patella. The medial retinacular incision was continued posteriorly, paralleling the inferior border of the VMO, until it reaches the medial intermuscular septum. Again, we preserved the rim of the tendon to ensure adequate suturing space following the transfer. When the medial intermuscular septum was visualized, the vastus medialis was released for a distance of 2–8 cm in an attempt to obtain a straight-line pull. The insertion of the vastus medialis was then transferred to the superior border of the patella.

**Fig. 3:**
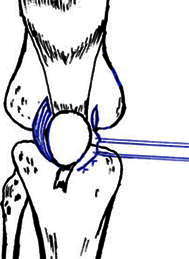
Releasing the lateral retinaculum and restoring the medial retinaculum

To release the lateral retinaculum of the patella, we increased the tension of the medial retinaculum by dividing it into two parts, and then used an imbricating stitch to link one part over the other, for 1.5 cm length, using No. 2 Ethibond sutures, while holding the patella in a normal position to attain 25 % lateral translation with the knee flexed 45°–60° (Fig. 3).

##### Procedure stage 2: lengthening the rectus femoris and vastus intermedius

**Fig. 4:**
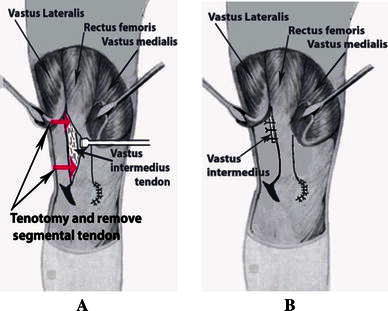
**a** Tenotomy and removing the segmental tendon of the vastus intermedius. **b** The remaining vastus intermedius being sutured beneath the rectus femoris

**Fig. 5:**
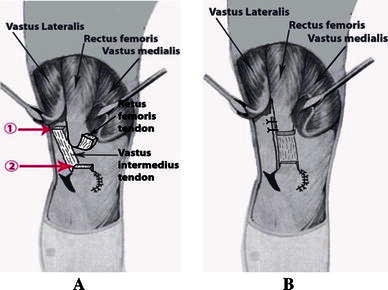
(*1*) Positional tenotomy at the musculotendinous junction of the vastus intermedius 5 cm above the patella. (*2*) Positional tenotomy of the rectus femoris 2 cm above the patella. **b** Two tendons, the vastus intermedius, and the rectus femoris being sewn together with knee flexion at 60°

We separated the adherence between the vastus intermedius tendon and the rectus femoris tendon. Next, we divided the vastus intermedius tendon at the musculotendinous junction, 5 cm above the patella. We performed knee flexion; if knee flexion >90° was achieved, the vastus intermedius tendon (5 cm above the patella) was removed (Fig. 4a), and the remaining vastus intermedius was sutured to the rectus femoris with a knee flexion of 60° (Fig. 4b) (variant 1). If knee flexion >90° was not achieved, the rectus femoris was detached 2 cm above the superior border of the patella, two tendons, the vastus intermedius, and the rectus femoris, being sewn together with using No. 2 Ethibond sutures while the knee was flexed at 60° (Fig. 5) (variant 2). Finally, the remaining vastus intermedius muscle was sutured to the rectus femoris.

##### Procedure stage 3: transfer of the VMO to the superior border of the patella

**Fig. 6:**
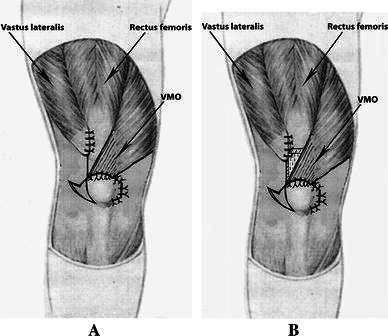
**a** The vastus medialis oblique (VMO) transferred to the superior border of the patella and the vastus lateralis sutured to the lateral side of the rectus femoris in variant 1. **b** The VMO transferred to the superior border of the patella and the vastus lateralis sutured to the joint tendons (the rectus femoris and the vastus intermedius) in variant 2

We transferred and sutured the inferolateral corner of the VMO to the superolateral pole of the patella, and then transferred and sutured the inferomedial corner of the VMO to the superomedial pole of the patella, using No. 2 Ethibond sutures with the knee flexed at 60°. The vastus lateralis was sutured to the lateral side of the rectus femoris (Fig. 6a) or to the joint tendon (the rectus femoris and the vastus intermedius) (Fig. 6b). The patella was then moved medially to check whether the release had been adequate (to ensure this, we checked that the patella was tracking normally and riding smoothly within the intracondylar groove, and that there was no tilting of the patella, nor excessive tension at the suture line). The knee was flexed and extended several times to see if the patella was tracking normally in its groove. At this stage, we expected that the patella should ride smoothly within the intracondylar groove and there should be no tilting of the patella or excessive tension at the suture line. It was also important to be certain that the lateral border of the patella was in line with the lateral border of the lateral femoral condyle.

#### Intraoperative biopsy

Biopsy specimens were taken of the muscular vastus lateralis, medialis, intermedius, rectus femoris, fascia lata, and lateral and medial retinaculum of the patella to find any potential tissue abnormalities, such as fibrosis or degeneration of the muscles or fibrous band.

#### Postoperative rehabilitation

**Fig. 7:**
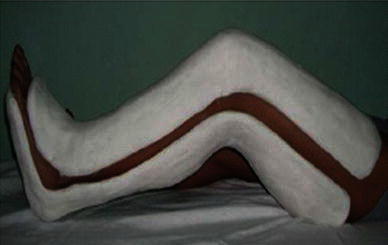
The double-capsule cast: a long-leg cast placed from above the knee down to the toes. The cast could be easily removed during physiotherapy for knee mobilization and has to replaced afterwards

Postoperatively, the knee was placed in a double-capsule cast with the knee flexed at 60°. This was a long-leg cast (Fig. 7). The cast could be removed easily for knee mobilization physical therapy and then be replaced afterwards. The patient was allowed out of bed 1 day after the operation. Static quadriceps exercises and gentle knee movements (passive range of motion not >60°, equal knee flexed at 60°, and the VMO was transferred and sutured to patella) were permitted, but more vigorous or extensive movements were discouraged during the first week, in order to minimize effusion or bleeding. In the first 2 weeks, a passive range of motion up to 60° and under 90° was performed with the physiotherapist. After 2 weeks, the range of motion was increased up to 90° (note: the limitations on the range of motion and weight-bearing counted only for the exercise periods, and outside of the exercise times, the cast had to be worn). After 3 weeks, the range of motion was increased to normal. The cast was removed after about 6 weeks, but without weight-bearing. Afterwards, patients were allowed to bear 25 % of their body weight for the first 2 weeks, and 50 % of their body weight in the following 2 weeks. Full weight-bearing was allowed between the fourth and sixth weeks after cast removal.

#### Follow-up

All patients were examined and evaluated by three other doctors, at follow-up times of 3, 6 weeks, 3, 6 months, 1 year, and afterwards every year. At final follow-up, after an average period of 4 years, 2 months (range 2 years, 7 months to 8 years, 4 months), the average patient age was 10 years, 9 months (range 9 years, 4 months to 15 years, 6 months).

We classified the results of the operation according to Kumar et al. [14], with objective evaluation undertaken using the functional knee-scoring systems of Fulkerson et al. [7] and Kujala et al. [15]. The scores were graded as follows: excellent: 90–100 points; good: 80–89 points; fair: 70–79 points; poor: <70 points.

### Statistical analysis

The data were analyzed with Epi Info 6.04 public domain statistical software for epidemiology, developed by the Centers for Disease Control and Prevention (CDC) in Atlanta, Georgia, USA (http://wwwn.cdc.gov/epiinfo/html/prevVersion.htm). We performed the χ^2^ test for percentages and Student’s *t*-test for the comparison of means. *p*-Values ≤0.05 were regarded as being statistically significant. All readings were provided as average values together with the appropriate standard deviation.

## Results

### Classification of patellar dislocation

Among our patient cohort, 38 knees (67.9 %) were type 1 and 18 knees (32.1 %) were type 2. After the surgery, we saw remarkable improvement in the clinical and roentgenographic findings in our patient cohort, as shown in Tables 1 and 2.

**Table 1:** Clinical signs before and after the operation

Signs	Preoperatively	Postoperatively	*p*-Value
Thigh atrophy	56 (100 %)	8 (14.3 %)	0.00001
The patellar medial glide test <5 mm at 30° of knee flexion	56 (100 %)	0	
Q-angle	14.8° (13.6°–21.7°)	8.2° (6°–13.1°)	0.00001

**Table 2:** Roentgenographic findings

Findings	Preoperatively	Postoperatively	*p*-Value
Patella alta	12 (21.4 %)	3 (5.4 %)	0.012525
Lateral patellar tilt	34 (60.7 %)	2 (3.6 %)	0.000000
Shallow patellar groove	16 (28.6 %)	2 (3.6 %)	0.000316
Flattening of the distal lateral femoral condyles	11 (19.6 %)	3 (5.4 %)	0.022271

The average preoperative Q-angle was 14.8° (range 13.6°–21.7°). There were three knees with a Q-angle >20°. Postoperatively, the average Q-angle was 8.2 °(range 6°–13.1°), which was significantly smaller, with a *p*-value = 0.0000001.

All patients had thigh atrophy preoperatively, which was related to the condition of fibrous muscle. There were 22 knees with corresponding atrophic thighs of 1.5 cm, all of which could be surgically corrected with procedure 2, variant 1. The remaining 34 knees had a thigh atrophy of 1.5–2.5 cm (2.5 cm in five thighs) and had to be surgically corrected according to procedure 2, variant 2. The recovery of thigh atrophy was highly significant, with a *p*-value = 0.00001.

Preoperatively, limited medial translation of the patella had been the result of slackness or contracture of the lateral patellar retinacula. After the intervention, the patella could be moved normally in both the superior–inferior and the medial–lateral directions at 20° of knee flexion. Preoperatively, four knees (7.1 %) had limited knee flexion of 95°–110°. Postoperatively, all four knees had no restriction in the range of motion.

Preoperatively, patella alta was apparent in 12 knees (21.4 %), and, postoperatively, it was only seen in three knees (5.4 %). Preoperatively, lateral patellar tilt was found in 34 knees (60.7 %), and postoperatively in only two knees (3.6 %), which was very significant, with a *p*-value of 0.00000. Preoperatively, there were shallow patellar grooves in 16 knees, of which 11 knees had flattening of the distal lateral femoral condyle. Postoperatively, only two knees (3.6 %) showed shallow patellar groove and three knees (5.4 %) showed flattening of the distal lateral femoral condyle. As can be seen from Table 2 above, one conclusion drawn is that, between pre- and postoperation, imaging roentgenography was improved significantly as time went by, with a *p*-value <0.05. Therefore, it is believed that the trochlear groove improvement was due to natural growth and development of the children.

### Pathological findings

Intraoperatively, the vastus lateralis muscles were markedly fibrosed, contractured, and shortened in all patients. This generally comprised a dense, fibrous band running along the muscle’s lower border. An abnormal attachment of the iliotibial tract to the patella was seen in all knees (100 %), with the iliotibial tract attached to the distal vastus lateralis muscle and the superolateral patella. This tract had a rolled anterior border and swept forward to the patella, rather than having its main part attached to the tibia; the lateral patella retinaculum was thick and fibrous. The insertion of the vastus lateralis into the upper pole of the patella was seen clearly, and fibrous tissue developed around its lateral aspect, distinct from the rest of the fascia lata. The iliotibial tract was attached to the muscular vastus lateralis at a location about 2.5–5 cm above the superolateral patella by fibrous tissues. Contractures were present in the rectus femoris in 34 knees (60.7 %) and in the vastus intermedius in 22 knees (39.3 %). The vastus medialis oblique was felt to be tight in 43 knees (76.8 %). The VMO was degenerated and straightened, and that affected its medial pulling function.

We performed muscular biopsies intraoperatively, which showed marked fibrosis of the muscular lateral vastus, intermedius vastus, and the lateral retinacular patella. The degeneration of the medial vastus and rectus femoris was also noted. All of these signs were also seen in the fibrosis of the quadriceps, triceps, gluteal, and deltoid muscles secondary to the antibiotic intramuscular injections. The quadriceps muscle was usually shortened due to a severe degree of fibrosis.

The pathological findings were well correlated with the severity of functional damages that needed the appropriate technique to correct (procedure stage 2, variant 1 or variant 2).

Postoperatively, all patients had the patella aligned with the patellar groove of the femur when flexing and extending. Knee function returned to normal in 47 knees (83.9 %). There was a limitation of flexion to 60°–90° in two knees (3.6 %) and >90° in three knees (5.3 %); loss of extension <10° in two knees (3.6 %) and 10°–20° in two knees (3.6 %); and no cases with a loss of extension of more than 20°. Loss of extension happened only when both the vastus intermedius and the rectus femoris were severely damaged (and surgically repaired with variant 2).

The differences in Kujala scores in the age groups ≤11 years and >11 years were both very significant, with *p*-values of 0.000001 and 0.000248, respectively. In the age group 9–11 years, the average patient age was 9 years, 11 months (range 9 years, 2 months to 10 years, 9 months). The mean Kujala score was 46.5 points (range 36–70) preoperatively and 91.2 points (range 76–100) at final follow-up. In the age group 11–15 years, the average patient age was 13 years, 11 months (range 12 years, 4 months to 14 years, 10 months). The mean Kujala score was 41.2 points (range 32–61) preoperatively and 95.2 points (range 87–100) at final follow-up. There were only two patients who were more than 15 years of age. The first of these two patients had an age at final follow-up of 15 years, 4 months, while the time of follow-up was 6 years, 4 months. This patient’s preoperative Kujala score was 49.4 and their postoperative Kujala score was 87 points. The second patient over 15 years old had an age at final follow-up of 15 years, 6 months, and the time of follow-up was 7 years, 9 months. That patient’s preoperative Kujala score was 52.1 and their postoperative Kujala score was 96 points.

Among all the knees, the mean Kujala score was 45.3 points (range 32–70) preoperatively and 92.4 points (range 76–100) at final follow-up.

The final follow-up showed excellent results in 39 knees (69.7 %), good results in 13 knees (23.2 %), fair results in four knees (7.1 %), and there were no poor results in any of the knees (see Table 4). An ugly scar was noted in nine knees (16.1 %).

Complications were as follows:

- Limitative knee movement: nine knees.
- Infections: none.
- Skin necrosis: none.
- Osteochondral patellar fracture: none.
- Redislocation or subluxation: none.

## Discussion

### Etiology and pathogenesis

Hnĕvkovský [4] and Miki as described by Gunn [5] were the first authors who described fibrous change after the intramuscular injection of antibiotic(s) in children in the 1960s. Since that time, most published cases of muscle contracture seem to be related to injections in childhood [16–22].

Our study found a history of repeated antibiotic intramuscular injections into the vastus lateralis muscle at the distal thigh in all patients, which caused thigh atrophy (see Table 1). The injection site correlates directly to the fibrotic region. Frasch and Saule [23] and McCloskey and Chung [24] proposed that, if long-term antibiotic treatment is anticipated in children, the intramuscular injection of antibiotic(s) should be avoided and the intravenous route be employed if possible, and we agree with their opinion.

Williams reported on 26 patients with stiffness of the knees, 13 of whom had experienced dislocation of the patella after intramuscular antibiotic injections [25]. Later, Alvarez et al. [26], Wenger et al. [27], Gunn [5], Mukherjee and Das [28], and Sharrard [29] also reported similar series. As previously described, our study found that all patients had a history of repeated antibiotic intramuscular injections into the quadriceps muscle, and, intraoperatively, we had observed the shortening and fibrosis of the quadriceps. In the majority of cases, the fibrosis had developed in the deep part of the vastus lateralis, the vastus intermedius, the fascia lata on the lateral side of the thigh, the lateral retinaculum, and superior aspects of the patella. The lesions and fibrosis had created an imbalance between the medial and lateral muscle forces on the patella, causing the thigh atrophy and leading to the lateral patellar dislocation. Thus, very probably, the patellar dislocation was secondary to the antibiotic injections in the past. In this condition, we agree with Williams’ opinion [25] that, if the patella is forcibly held in the midline, it is impossible to flex the knee more than about 30°. Further flexion is then possible only if the patella is allowed to dislocate, when a full range of motion is readily obtainable. In this type of dislocation, the contracture was the primary pathology, whereas medial laxity or weakness of the medial stabilizers of the patella was secondary.

In our study, we found knee abnormalities including lateral patella tilt in 34 knees (60.7 %), patella alta in 12 knees (21.4 %), shallow patellar groove in 16 knees (28.6 %), and flattening of the distal lateral femoral condyles in 11 knees (19.6 %) (see Table 2), which can be explained by the mechanical damage due to the shortening of the muscle leading to abnormal forces on growing bone and the frequent patellar dislocation.

In our hospital, from October 1984 to December 2004, we recognized 278 cases of quadriceps contracture, 34 cases of triceps contracture, 12 cases of gluteal muscle contracture, 182 cases of fibrous deltoid muscle [30], 38 cases of fibrous long head of the triceps [31], and 161 cases of fibrous rectus femoris muscle [32].

### Surgical technique

Paletta, in 1820, reported the first case of congenital dislocation, followed by Wuhzer in 1835, Lelius in 1840, and Michaelis in 1854, as described Conn [33]. In 1959, Cotta [34] counted 137 surgical methods directed at solving the problems of the unstable patella; however, variations in reporting and study design make comparisons among these studies almost impossible. Many reconstructive procedures for recurrent dislocation of the patella have been described. Most of them concentrate on the medial stabilization of the patella or its tendon. The tibial tubercle shift of Roux in 1888 [35] was popularized by Hauser in 1938 [1], medial transplantation of the lateral half of the patellar tendon was described by Goldthwait in 1904 [36], and the semitendinosus tenodesis of Galeazzi in 1922 [37] was popularized by Baker et al. in 1972 [2]. Reconstruction of the extensor mechanism was reported by Hughston in 1972 [38] and vastus medialis transplantation, distally and laterally to the front of the patella, was suggested by Madigan et al. in 1975 [3]. There was also the Elmslie–Trillat procedure of the lateral retinacular release, the medial retinacular plication and medial transfer of the tibial tubercle reported by Cox in 1976 [39], and arthroscopic lateral release with or without medial plication (McGinty and McCarthy [40]; Metcalf [41]). Ober suggested transferring an Iliotibial tract passed beneath the aponeurosis and suturing it to the medial aspect of the tibia in 1935 [42]. All authors agreed that, in children with immature bones, the intervention should be done only on the soft tissues. In 1975, Madigan et al. [3] (followed by other authors subsequently) transferred the VMO laterally and distally, and sutured it directly to the anterior side of the patella or to the medial rectus femoris tendon, as per West and Soto-Hall [43], hoping to stabilize the patella and direct force on it medially, thereby hoping to prevent lateral displacement.

### Anatomy

The vastus medialis muscle can be functionally divided into long and oblique components. Lieb and Perry [44] reported that, in the larger and proximal long component, the fibers have a more vertical orientation, deviating medially 15°–18° from the longitudinal axis of the femur, while the fibers in the distal quarter of the muscle, the oblique component, deviate 50°–55° from the femoral axis.

Furthermore, the vector of pull of the oblique component is similar to that of the long component. The only unique function of the VMO is to exert a medially directed force to the superior border of the patella and, thereby, prevent lateral displacement. The impression is supported by the findings of Lieb and Perry [44].

In our study, the VMO was commonly severely atrophic and more vertically oriented rather than oblique, as reported by Andrish [45]. To complete our first goal, which was to restore the original direction force of the VMO, we released it from the medial intermuscular septum and transferred it to the superior border of the patella. Unlike Madigan et al.’s technique, in which they transferred the insertion of the vastus medialis laterally and distally to overly the patella, in our patients, the vastus medialis was tight and shortened, so it could not be transferred distally and laterally to the front of the patella. In these cases, if the VMO was sutured to the front of the patella, it would badly limit the knee flexion.

Our second goal was to restore the normal alignment and function of the extensor mechanism. We released the insertion of the vastus lateralis to the patella and iliotibial tract, so that the lateral forces on the patella were reduced. The vastus intermedius and rectus femoris also limited the movement of the knee. We lengthened the vastus intermedius and rectus femoris, with the goal of improving flexion of the knees. However, this process should be undertaken step by step: if a patient’s knee flexion of more than 90° could be achieved with vastus intermedius release only, then we performed the operation according to procedure 2, variant 1; if not, we employed variant 2 (see Table 3).

**Table 3:** Main contracture, associated contracture, and their relationship with the surgical technique and knee functions

Main contracture	Associated contracture	Surgical technique	Function of knee movement					
			Normal	Limitation of flexion		Loss of extension		
				60° to ≤90°	>90°	≤10°	>10° to ≤20°	>20°
Vastus lateralis, *n* = 22	Vastus intermedialis	Variant 1	20	0	2	0	0	0
Vastus lateralis, *n* = 34	Vastus intermedialis and rectus femoris	Variant 2	27	2	1	2	2	0
			47 (83.9 %)	2 (3.6 %)	3 (5.3 %)	2 (3.6 %)	2 (3.6 %)	0

Although we did not address the ligamentum patellae in our surgical procedures, the patella alta still improved. These procedures can be classified into soft-tissue balancing procedures and bony transfer procedures. In the skeletally immature patient and with open growth plates, transfer of the tibial tubercle must be avoided, if possible, to prevent premature physeal closure and subsequent development of genu recurvatum. In children, soft-tissue balancing procedures are widely accepted methods of treatment, such as the surgical procedure which involves a lateral retinacular release with mobilization of the vastus medialis muscle to a more distal and lateral position on the extensor mechanism. In this study’s surgical technique, only muscular and ligament components were restored, as the bony interventions were not recommended in children. The patella alta was improved by growth and development. We also believe that the trochlear groove improvement was due to natural growth and development of the children (see Table 4).

**Table 4:** Age of the patients at follow-up and Kujala scores

Age of patient	Final follow-up				Total	Kujala score (avg) (preop)	Kujala score (avg) (postop)	*p*-Value
	Excellent	Good	Fair	Poor				
>9 to ≤11 years	17 (43.6 %)	3 (23.1 %)	3 (75.0 %)	0	23 (41.1 %)	46.5	91.2	0.00001
>11 to ≤15 years	21 (53.8 %)	9 (69.2 %)	1 (25.0 %)	0	31 (55.3 %)	41.2	95.2	0.00024
>15 years	1 (2.6 %)	1 (7.7 %)	0	0	2 (3.6 %)	49.4^a^	87^a^	
						52.1^a^	96^a^	
Totals	39 (69.7 %)	13 (23.2 %)	4 (7.1 %)	0	56	45.3	92.4	

^a^Preop and postop Kujala scores for the >15 years age group were individual values for two individuals, not averages. All other Kujala scores are averages

### Limitations and complications

#### Loss of extension

In 34 knees on which we had operated with variant 2, four knees had extension loss of under 20°. This result was due to the postoperative quadriceps weakness. Although that complication rate was low, and happened in severely damaged quadriceps, we still suggest that quadriceps lengthening should only be considered when the vastus intermedius release alone cannot attain a knee flexion of more than 90°.

#### Limitation of flexion

Limited knee flexion was seen in five knees (two knees with variant 1 and three knees with variant 2). All knees had severely fibrous vastus intermedius and degeneration of the rectus femoris during pathological examination. In our opinion, this may be due to two causes, either (1) incomplete liberation of the surrounding fibrosis or (2) inadequate lengthening of the quadriceps. Therefore, we suggest that, before finalizing the operation, knee flexion should be performed. If knee flexion >90° was not achieved, the quadriceps should be further lengthened.

## Conclusions

In children, an unstable patella with stiffness of the knee may occur after repeated antibiotic intramuscular injections, which causes fibrosis in the quadriceps. Mechanical damage due to shortening of the muscle lead to abnormal forces on growing bone, and the frequent patellar dislocation, in turn, causes anatomical lesions, such as patellar tilt, patella alta, shallow patellar groove, and flattening of the distal lateral femoral condyles.

In surgery for dislocation of the patella, it is difficult to obtain a normal position of the patella in children using soft-tissue balancing procedures alone. In our cases of pediatric dislocation of the patella caused by iatrogenic quadriceps fibrosis, the three-part surgical procedure combining capsulorrhaphy, quadricepsplasty, and transfer of the vastus medialis oblique (VMO) to the superior border of the patella showed great success in restoring the realignment mechanism of the patella. The technique is simple, safe, and effective in skeletally immature children.
